# Association of Social Relationships and Depression Among Older Mexican Adults: Does a Rural or Urban Context Matter?

**DOI:** 10.1093/geroni/igab046.1938

**Published:** 2021-12-17

**Authors:** Paige Downer, Rebeca Wong

**Affiliations:** University of Texas Medical Branch, Galveston, Texas, United States

## Abstract

Social characteristics such as strong community and family ties have been associated with positive mental health outcomes in older adults. However, this evidence is based primarily on non-Hispanic White populations and may vary according to living in a rural versus urban community. We hypothesize that the positive impact of available social networks, perceived support, and social participation on older Mexican adults’ likelihood for high depressive symptoms (i.e., depression) will be greater for those living in rural (community < 2,500 people) than urban communities. Data came from the 2012 Wave of the Mexican Health and Aging Study. Depressive symptoms were measured using a 9-item version of the Center for Epidemiologic Studies Depression Scale. Social participation is the respondent’s self-reported participation in hobbies, religious activities, volunteering, and visits with neighbors. Available social network is measured as having relatives and/or good friends living in the neighborhood. Perceived support is the respondent’s perception of friends/family’s willingness to help with finances and personal care. The final sample of 6,266 respondents was majority (62.4%) female, mean age of 69 years, 17.8% lived in a rural community, and 34.5% with depression. Logistic regression models stratified by rural/urban indicated that available social network and perceived social support were not associated with depression in rural or urban communities. In general, the social participation activities were associated with significantly lower odds of depression for older adults living in urban but not rural communities. This research highlights the influence of older adults’ community on their social relationships and mental health.

